# Knowledge on Fetal Alcohol Syndrome in Slovak Population

**Published:** 2019-03

**Authors:** Pavol BENO, Ingrid JUHASOVA, Martin SAMOHYL

**Affiliations:** 1. Department of Laboratory Medicine, Faculty of Health Sciences and Social Work, Trnava University, Trnava, Slovakia; 2. Department of Health Studies, University of Polytechnics Jihlava, Jihlava, Czech Republic; 3. Institute of Hygiene, Faculty of Medicine, Comenius University in Bratislava, Bratislava, Slovakia

## Dear Editor-in-Chief

High alcohol intake (over 48–60 gr. ethanol/day) during pregnancy may cause fetal alcohol syndrome (FAS). The criteria of FAS diagnosis are prenatal alcohol consumption, newborn’s growth retardation, newborn’s characteristic facial features and newborn’s neurological abnormalities (1). The exposure to alcohol before birth can cause developmental disabilities and birth defects known as fetal alcohol spectrum disorders. Fetal alcohol spectrum disorders can cause certain pregnancy problems (miscarriage), premature birth and stillbirth. It is recommended not to drink any alcohol during pregnancy. If a child’s fetus is not exposed to alcohol before birth, risk of fetal alcohol spectrum disorders is not possible (2). The global prevalence of FAS is 14.6% per 10000 people (3). Fetal alcohol syndrome is economic burden for a society. Several pregnancy nutrition studies (4, 5) were found, while studies about FAS knowledge are limited.

In this study, we aimed to analyse the awareness of FAS in Slovak population.

The standardized questionnaire “Fetal Alcohol Syndrome Survey” (FASS) was used (6). The questionnaires collection was carried out from 7/2016 to 9/2016. Overall, 420 completed questionnaires were collected. An average age of the study sample was 29.3±8.2 yr (n=95, 22.6% of males).

About, 78% of the respondents did not hear about FAS. 82.9% of the respondents agreed with the statement that drinking alcohol during pregnancy increases the risk of mental retardation for a fetus. Most respondents (81.6%) claimed that drinking alcoholic beverages during pregnancy can be a cause of the miscarriage. 71.2% of respondents agreed with the statement that drinking alcohol during pregnancy increases the risk of a low birth weight. The respondents agreed (84.5%) that drinking alcoholic beverages during pregnancy increases the risk of birth defects. In a part of the questionnaire intended only for pregnant females, 55% of females, alcoholic beverages during their pregnancy were offered them by someone else. Overall, 60% of females refused specific kind of alcohol, 37% of females had alcohol in a symbolical way, 2% of females could not answer and 1% of females drank alcohol. Greater awareness of FAS was found in females than males (*P*<0.001). Differences in age and education of the respondents in terms of awareness are significant (*P*<0.001).

Greater awareness of FAS was found in the older age respondents and in the respondents with a higher degree of education. More effective alcohol drinking prevention strategies during pregnancy are urgently needed.
